# Recombinant Leucine-Rich Repeat Flightless-Interacting Protein-1 Improves Healing of Acute Wounds through Its Effects on Proliferation Inflammation and Collagen Deposition

**DOI:** 10.3390/ijms19072014

**Published:** 2018-07-10

**Authors:** Zlatko Kopecki, Natalie E. Stevens, Gink N. Yang, Elizabeth Melville, Allison J. Cowin

**Affiliations:** Regenerative Medicine, Future Industries Institute, University of South Australia, Adelaide SA 5095, Australia; natalie.stevens@unisa.edu.au (N.E.S.); gink.yang@unisa.edu.au (G.N.Y.); elizabeth.melville@unisa.edu.au (E.M.); allison.cowin@unisa.edu.au (A.J.C.)

**Keywords:** LRRFIP-1, flightless I, wound healing, collagen

## Abstract

Wound healing is an increasing clinical problem involving substantial morbidity, mortality, and rising health care costs. Leucine-rich repeat flightless-interacting protein-1 (LRRFIP-1) regulates toll-like receptor (TLR)-mediated inflammation, suggesting a potential role in the healing of wounds. We sought to determine the role of LRRFIP-1 in wound repair and whether the exogenous addition of recombinant LRRFIP-1 (rLRRFIP-1) affected healing responses. Using a model of full-thickness incisional acute wounds in BALB/c mice, we investigated the effect of wounding on LRRFIP-1 expression. The effect of rLRRFIP-1 on cellular proliferation, inflammation, and collagen deposition was also investigated. LRRFIP-1 was upregulated in response to wounding, was found to directly associate with flightless I (Flii), and significantly increased cellular proliferation both in vitro and in vivo. rLRRFIP-1 reduced Flii expression in wounds in vivo and resulted in significantly improved healing with a concurrent dampening of TLR4-mediated inflammation and improved collagen deposition. Additionally, decreased levels of TGF-β1 and increased levels of TGF-β3 were observed in rLRRFIP-1-treated wounds suggesting a possible antiscarring effect of rLRRFIP-1. Further studies are required to elucidate if the mechanisms behind LRRFIP-1 action in wound repair are independent of Flii. However, these results identify rLRRFIP-1 as a possible treatment modality for improved healing of acute wounds.

## 1. Introduction

The leucine-rich repeat flightless-interacting protein-1 (LRRFIP-1) protein was originally discovered for its interactions with an actin-remodeling protein called flightless I (Flii) [1]. Flii is a negative regulator of wound repair via its effect on cellular processes affecting wound inflammation and collagen synthesis [2,3]. However, the role of LRRFIP-1 in this context has yet to be investigated. LRRFIP-1 is widely expressed in fetal tissues, has low expression in normal adult skin, and is mainly localized to the nucleus and the actin cytoskeleton [4]. Previous reports pertaining to the function of LRRFIP-1 have focused on its regulation of the Wnt/β-catenin canonical transcription pathway [5], which has been described as critical for proliferation of mesenchymal fibroblasts during wound repair [6]. Studies have suggested that increased LRRFIP-1 levels may affect cell behavior, and high levels of LRRFIP-1 have been observed in liver and lung cancers, with LRRFIP-1 being shown to promote cancer metastasis and cancer cell invasion. Silencing of LRRFIP-1 reverses the epithelial-mesenchymal transition via its effects on the Wnt/β-catenin signaling pathway, while its knockdown in liver cancer cells reduces cell growth and increases apoptosis [7,8]. These studies suggest that altering LRRFIP-1 levels may affect cellular responses important during proliferative and remodeling stages of wound repair. Additionally, LRRFIP-1 has been identified as a biological DNA sensor required for the production of interferon-beta (IFN-β), and the transient knockdown of LRRFIP-1 results in a 50% reduction of IFN-β secretion by infected peritoneal macrophages, while the stable knockdown in RAW 264.7 cells suppressed IFN-β production by 80% via the β-catenin-dependent pathway [9], suggesting another possible role in regulating tissue inflammation. Indeed, IFN-β has been shown to regulate proliferation of many cell types important within a wound including the endothelium and has inhibitory effects on wound angiogenesis [10]. 

LRRFIP-1 protein positively modulates toll-like receptor 4 (TLR4)-mediated innate inflammatory responses and subsequent cytokine secretion, both of which are important in early healing of acute wounds [11]. Additionally, LRRFIP-1 acts as a transcriptional activator via its interplay with Flii and regulates beta-catenin dependent transcription [4]. LRRFIP-1 has also been identified as a TNF-α repressor [4,12,13], leading to decreased NF-κB activation and altered M1 and M2 macrophage populations, suggesting potential effects on wound repair and matrix synthesis [14]. In addition, LRRFIP-1 competition with Flii for the myeloid differentiation primary response gene 88 (MyD88) binding sites, a known adaptor protein mediating TLR signaling, actively promotes NF-κB activity in Lipopolysaccharide (LPS)-stimulated HEK293 cells and macrophage cell lines, suggesting that LRRFIP-1 can positively regulate TLR signaling [11]. Together, these studies highlight an important role for LRRFIP-1 in the regulation of inflammatory signaling pathways known to regulate wound healing outcomes. However, to date, no studies have examined the role of LRRFIP-1 in wound repair. Here, we investigated the effect of wounding on LRRFIP-1 expression and the effect of recombinant LRRFIP-1 (rLRRFIP-1) on the healing of incisional wounds.

## 2. Results

### 2.1. LRRFIP-1 Is Upregulated in Response to Wounding

**Figure 1:**
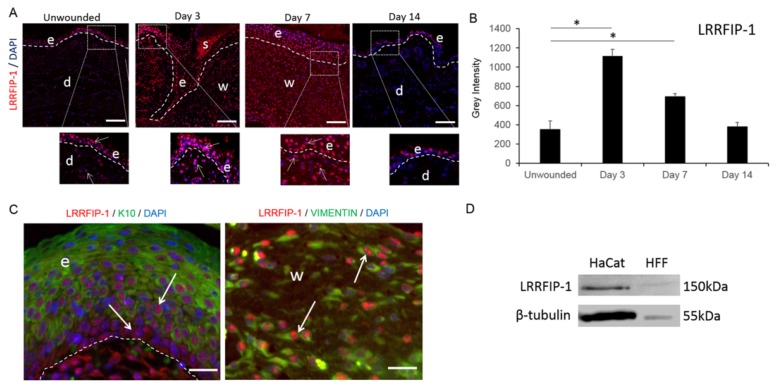
Leucine-rich repeat flightless-interacting protein-1 (LRRFIP-1) expression is upregulated in response to wounding. (**A**) Immunohistochemistry for LRRFIP-1 (red) was performed on unwounded and wounded BALB/c mice skin. Representative images are shown for 0, 3, 7, and 14 day wounds. In each image, the epidermis is delineated by white dotted line, e = epidermis, s = scab, and w = wound area, d = dermis. Magnification ×20. Insert ×40. White arrows = LRRFIP-1 positive cells. Scale Bar = 50 µm; (**B**) Graphical representation of dermal LRRFIP-1 protein expression in BALB/c mice skin at day 0, 3, 7, and 14 post-wounding. Results represent mean ± SEM. * *p* < 0.05. Figure is representative of two independent experiments (*n* = 10); (**C**) Immunohistochemistry for LRRFIP-1 (red), keratin-10 (green), vimentin (green), and 4′,6-diamidino-2-phenylindole (DAPI) (blue) was performed on day 3 wounds of mice skin. White arrows = LRRFIP-1 positive cells. Magnification ×60. Scale Bar = 30 µm; (**D**) Keratinocyte (HaCaTs) and fibroblast (HFFs) cell lysates were prepared from sub-confluent in vitro cultures and immunoblotted with LRRFIP-1 antibody and β-tubulin loading control.

In unwounded adult BALB/c mice skin, LRRFIP-1 expression was low and mainly observed in the nucleus of keratinocytes within the epidermis (Figure 1A). Wounding significantly increased LRRFIP-1 expression in both the dermis and epidermis (Figure 1A,B) and co-staining of LRRFIP-1 with keratinocyte (keratin-10) and fibroblast (vimentin) cell markers at day 3 post-wounding showed that both keratinocytes and fibroblasts expressed LRRFIP-1 in response to wounding. LRRFIP-1 was observed to be predominantly nuclear in localization in keratin-10-positive keratinocytes, although some cytoplasmic staining was observed in keratin-10-negative basal keratinocytes (Figure 1C). LRRFIP-1 was observed in the nucleus of vimentin positive fibroblasts within the wound bed (Figure 1C) and LRRFIP-1 was detected in cell lysates of the human keratinocyte cell line (HaCaTs) and human foreskin fibroblasts (HFFs) using Western blotting (Figure 1D), suggesting that both keratinocytes and fibroblasts produce LRRFIP-1. A temporal effect of wounding on LRRFIP-1 localization was observed with significantly increased LRRFIP-1 levels at day 3 and day 7 post-wounding vs. unwounded skin returning to basal levels by day 14 post-wounding (Figure 1A,B).

### 2.2. rLRRFIP-1 Increases Cell Proliferation and Improves Wound Healing

**Figure 2:**
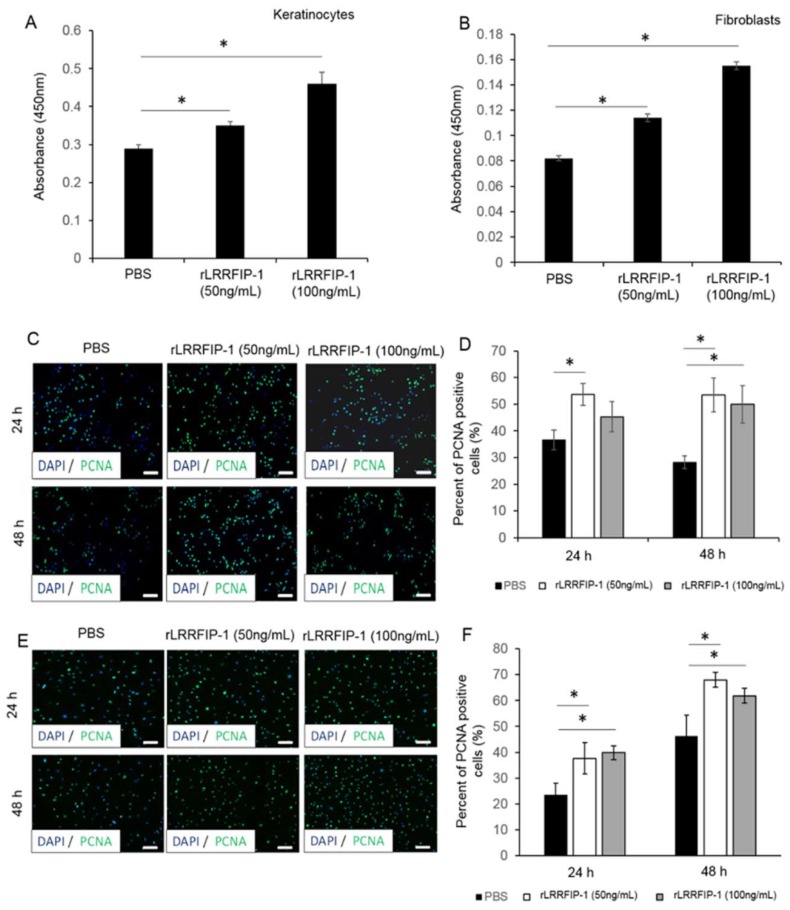
Recombinant LRRFIP-1 (rLRRFIP-1) increases cell proliferation in vitro. (**A**,**B**) HaCats and HFFs treated with rLRRFIP-1 (50 ng/mL and 100 ng/mL) and PBS control were cultured and the effect on proliferation determined at 24 h using WST-1 assay. *n* = 6. Subconfluent primary mouse keratinocytes (**C**,**D**) and fibroblasts (**E**,**F**) were treated with rLRRFIP-1 (50 ng/mL and 100 ng/mL) and PBS control and the effect on cell proliferation was analysed using costaining of proliferating cell nuclear antigen (PCNA) (green) and DAPI (blue) at 24 and 48 h post-treatment. *n* = 6. Magnification ×20. Scale Bar = 100 µm. Results represent mean ± SEM. * *p* < 0.05.

**Figure 3:**
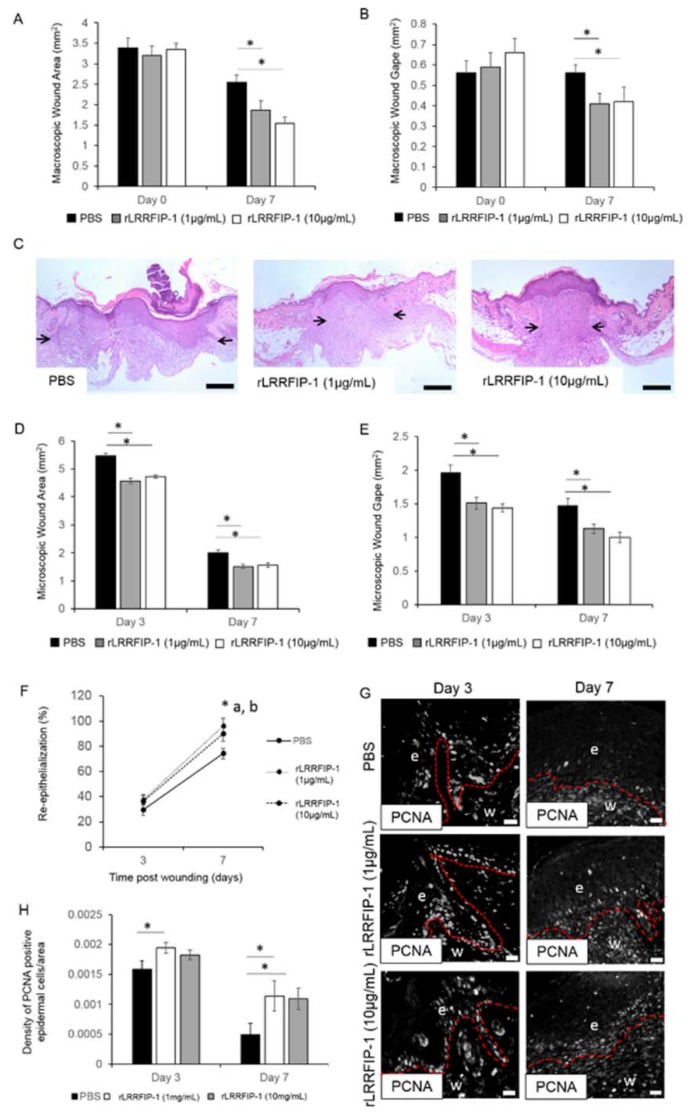
rLRRFIP-1 improves wound healing and cell proliferation in vivo. Two full-thickness, 1-cm incisions were made through the dorsal skin of wild-type (WT) mice. Intradermal injections of rLRRFIP-1 (1 and 10 µg/mL) and PBS control were administered at day 0 at wound margin (*n* = 10). Wounds were harvested at day 7 post-wounding. (**A**,**B**) Graphical representation of macroscopic analysis of wound area and wound gape at days 0 and 7 post-wounding; (**C**) Representative haematoxylin and eosin stained 7-day wound sections for mice wounds treated with rLRRFIP-1 (1 µg/mL and 10 µg/mL) and PBS control illustrating differences in wound healing outcomes. Black arrows highlight the differences in wound gape between different treatment groups; (**D**,**E**) Graphical representation of microscopic analysis of wound area and wound gape at day 7 post-wounding; (**F**) Wound re-epithelialization was evaluated by measuring the percentage of the wound that had epidermal covering at days 3, 7, and 14 post-wounding. * a = rLRRFIP-1 (1 µg/mL) vs. PBS; * b = rLRRFIP-1 (10 µg/mL) vs. PBS. Results represent means ± SEM. *n* = 10; (**G**,**H**) Representative images of PCNA-positive epidermal cells (white fluorescence) in the epidermal tongue of the re-epithelializing day 3 and day 7 wild-type wound treated with rLRRFIP-1 (1 or 10 µg/mL) or PBS control and graphical representation of PCNA-positive cells counted in the neoepidermis and expressed as cell density/area. Red dotted line represents the dermal–epidermal junction. e = epidermis, w = wound. Magnification ×20. Scale Bar = 20 µm. Results represent mean ± SEM. * *p* < 0.05. Figure is representative of two independent experiments (*n* = 10).

The effect of rLRRFIP-1 on cellular activity was determined using both a metabolic assay (WST-1) and by staining cells for the proliferating cell nuclear antigen (PCNA) marker. The addition of rLRRFIP-1 (50 and 100 ng/mL) to human keratinocytes (HaCaTs) and human foreskin fibroblasts (HFFs) led to a dose-dependent increase in metabolic activity compared to Phosphate-buffered saline (PBS)-treated controls (Figure 2A,B). Additionally, primary mouse keratinocytes and mouse fibroblasts treated with rLRRFIP-1 (50 and 100 ng/mL) showed a significant increase in PCNA staining at 24 and 48 h post-treatment (Figure 2C,F). Together, these results suggest LRRFIP-1 can affect cellular processes, including proliferation (Figure 2A,F). Incisional wounds on mice were intradermally treated with rLRRFIP-1 (1 and 10 µg/mL) and the effect on healing determined. Macroscopic wound measurements of wound area and wound gape showed that rLRRFIP-1 (1 and 10 µg/mL) improved healing, with wounds being significantly smaller and more contracted at day 7 post-injury (Figure 3A,B). No difference was observed between the two doses tested (1 or 10 µg/mL) (Figure 3A,B). Similar observations were noted following microscopic histological analysis of wound area and wound gape at days 3 and 7 post-wounding, with a significant improvement in healing observed following rLRRFIP-1 treatment at both the high and low dose (1 and 10 µg/mL) when compared to PBS-treated control wounds (Figure 3C,E). Additionally, analysis of wound re-epithelialization showed a significant improvement at day 7 post-wounding following rLRRFIP-1 treatment, irrespective of tested dose when compared to PBS-treated control wounds (Figure 3F). The effect of rLRRFIP-1 treatment (1 and 10 µg/mL) on keratinocyte proliferation was confirmed in vivo with significantly increased numbers of PCNA-positive keratinocytes observed in the neoepidermis of rLRRFIP-1-treated wounds both at days 3 and 7 post-wounding (Figure 3G,H). Contrary to the observations in vitro, PCNA staining of dermal wound fibroblasts showed no effect on cell proliferation in vivo. Based on no significant differences observed between the two treatment doses, the remainder of the analysis focused on wounds treated with the lower-dose (1 µg/mL) rLRRFIP-1 treatment.

### 2.3. LRRFIP-1 Directly Associates with Flii Reducing Its Activity in Wounds In Vivo

**Figure 4:**
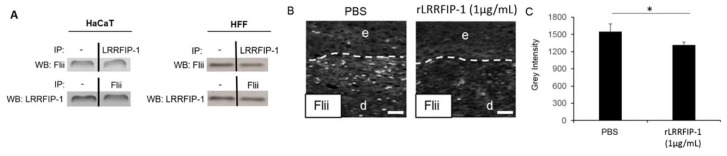
LRRFIP-1 directly associates with flightless I (Flii) and reduces its levels in wounds in vivo. (**A**) Anti-LRRFIP-1 and anti-Flii immunoprecipitates (IP) were prepared from confluent HaCaTs and HFFs and immunoblotted with Flii and LRRFIP-1 antibodies, respectively. (**B**,**C**) Representative images and graphical analysis of Flii expression (white fluorescence) in LRRFIP-1-treated (1 µg/mL) wild-type (WT) mouse wounds at day 7 post-wounding. In each image, the epidermis is delineated by white dotted lines, e denotes epidermis, and d denotes wounded dermis area. Magnification ×20. Scale Bar = 50 µm. * *p* < 0.05. Figure is representative of two independent experiments (*n* = 10).

In order to investigate if LRRFIP-1 associates with Flii during wound repair, immunoprecipitates and cell lysates from wounded HaCaTs and HFFs were analysed using Western blotting. LRRFIP-1 directly coimmunoprecipitated with Flii (Figure 4A). The ability of rLRRFIP-1 to alter Flii levels in wounds in vivo was investigated with rLRRFIP-1 (1 µg/mL) treatment, resulting in decreased Flii levels in day 7 wounds in vivo (Figure 4B). 

### 2.4. Inflammation Is Decreased in rLRRFIP-1-Treated Wounds

**Figure 5:**
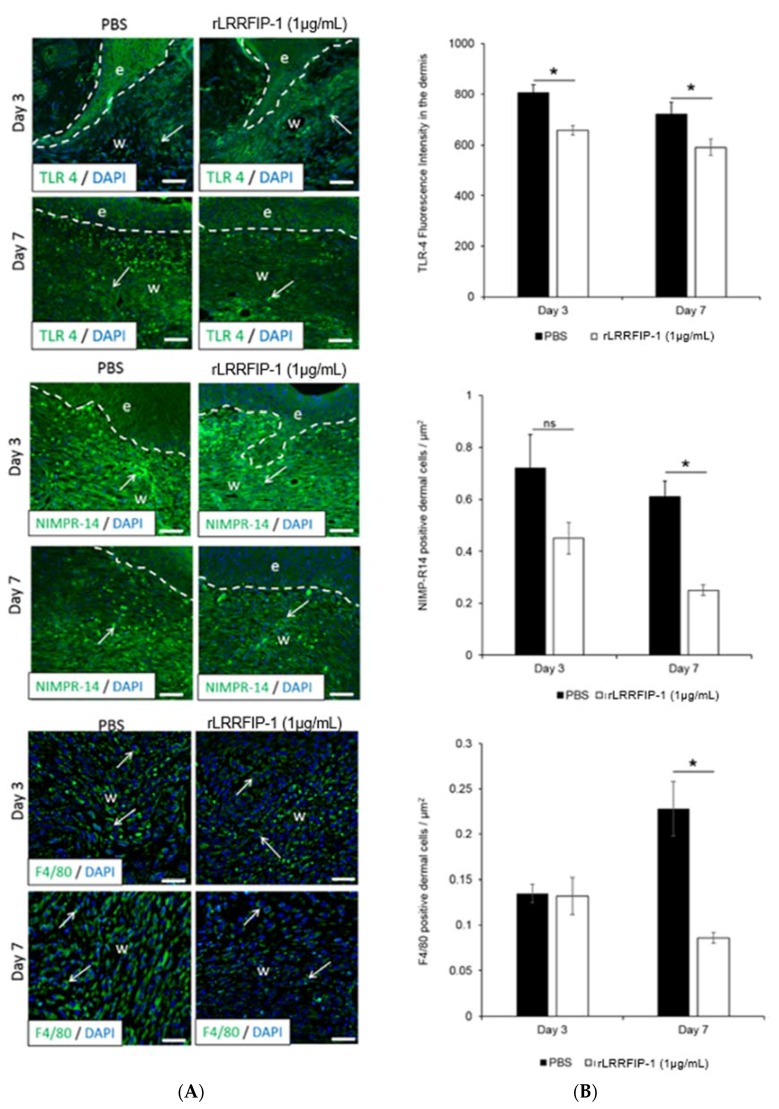
rLRRFIP-1 treatment modulates toll-like receptor 4 (TLR4) signalling and reduces dermal wound inflammation in vivo. (**A**) Representative images of TLR4, NIMP-R14, and F4/80 positive dermal cells (green fluorescence) in rLRRFIP-1 (1 µg/mL) or PBS-control-treated wounds at day 7 post-healing illustrating decreased inflammatory response following rLRRFIP-1 treatment. Epidermis is delineated by white dotted lines, e denotes epidermis, w denotes wound, and white arrows show positive stained cells. Magnification ×20. Scale Bar = 50 µm applies to all images; (**B**) Graphical analysis of TLR4 signaling and NIMP-R14 and F4/80 positive dermal cells in wounds of rLRRFIP-1 or PBS-treated animals. Results represent mean ± SEM. * *p* < 0.05. ns = not significant. *n* = 10.

To determine the cellular processes underpinning the improved healing responses observed in rLRRFIP-1-treated mouse wounds, the effect of rLRRFIP-1 (1, 10 µg/mL) on wound inflammation and angiogenesis was investigated. Compared to PBS-control-treated wounds, the day 3 and day 7 wounds of mice treated with rLRRFIP-1 (1 µg/mL) showed significantly decreased Toll-like Receptor 4 (TLR4) expression with a concurrent decrease in number of infiltrating neutrophils (NIMP-R14-positive) and macrophages (F4/80-antigen-positive cells) (Figure 5A,B). Treatment of wounds with the higher dose of rLRRFIP-1 (10 µg/mL) had the same effect as rLRRFIP-1 at 1 µg/mL (Supplementary Figure S2). rLRRFIP-1 treatment (1 µg/mL or 10 µg/mL) had no effect on wound angiogenesis, with similar dermal expression of CD31 observed at day 3 and day 7 wounds treated with rLRRFIP-1 or PBS control (Supplementary Figure S1). 

### 2.5. Treatment of Acute Wounds with rLRRFIP-1 Affects TGF-β Signalling and Collagen Deposition in Wounds In Vivo

**Figure 6:**
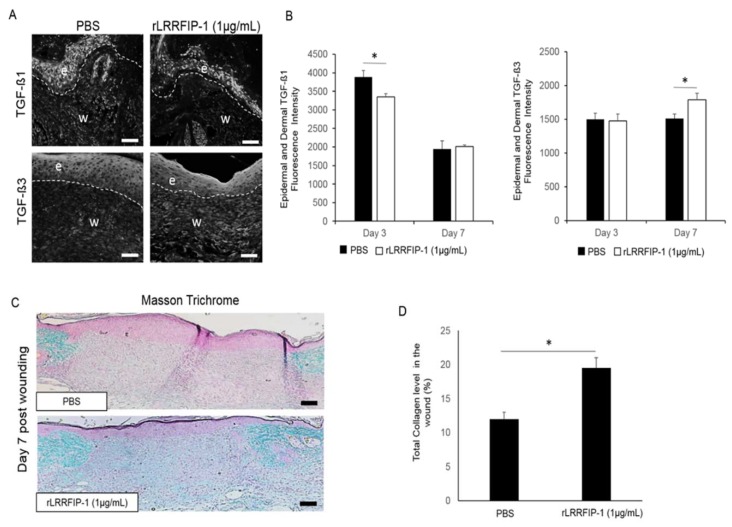
rLRRFIP-1 treatment affects TGF-β levels and early collagen deposition in wounds in vivo. (**A**,**B**) Representative images and graphical analysis of TGF-β1 and TGF-β3 levels (white fluorescence) in day 3 and day 7 wounds treated with rLRRFIP-1 (1 µg/mL) or PBS control illustrating decreased TGF-β1 and increased TGF-β3 levels in wounds in vivo following rLRRFIP-1 treatment. Epidermis is delineated by white dotted lines, e denotes epidermis, and w denotes wound dermis. Magnification ×20. Scale Bar = 50 µm; (**C**,**D**) Representative images and graphical analysis of total collagen levels in day 7 wounds in vivo post-treatment with rLRRFIP-1 (1 µg/mL) or PBS control illustrating increased total collagen deposition in wounds (green) following treatment with rLRRFIP-1 (1 µg/mL). Magnification ×10. Scale Bar = 100 µm. Results represent mean ± SEM. * *p* < 0.05. *n* = 10.

To better understand the effect of rLRRFIP-1 treatment on wound healing, mouse wounds treated with rLRRFIP-1 (1 µg/mL) or PBS control were examined for TGF-β signaling (day 3 and day 7 post-wounding) and total collagen deposition (day 7 post-wound healing). rLRRFIP-1-treated wounds showed significantly decreased levels of pro-scarring TGF-β1 expression in both the neoepidermis and dermal matrix at day 3 post-wounding (Figure 6A,B). No significant difference in TGF-β3 levels was observed at day 3 post-wounding irrespective of treatment, however, TGF-β3 levels were significantly increased in the dermis of rLRRFIP-1- treated (1 µg/mL) wounds at day 7 post-wounding (Figure 6A,B). The rLRRFIP-1 treatment was also shown to affect collagen deposition, with Masson’s trichrome staining for collagen levels revealing a >40% increase in total collagen levels in day 7 wounds of rLRRFIP-1- treated (1 µg/mL) mice (Figure 6C,D). Treatment of wounds with a higher dose of rLRRFIP-1 (10 µg/mL) did not result in a further increase in collagen synthesis. (Supplementary Figure S3). 

## 3. Discussion

LRRFIP-1 is a known modulator of the innate inflammatory responses including TLR signaling and cytokine secretion and has been shown to play an important role in cellular processes involved in cancer cell invasion [8,9,11]. However, its function in wound healing has not been investigated to date. We now show that LRRFIP-1 is mainly produced by keratinocytes in the unwounded skin but is upregulated in both keratinocytes and fibroblasts in response to wounding, suggesting a role in the wound repair process. LRRFIP-1 expression peaks at day 3 postinjury, which is suggestive of a potential role for LRRFIP-1 during the inflammatory phase of wound repair. Additionally, we show that rLRRFIP-1 can affect cellular proliferation both in vitro and in vivo, which may directly affect wound re-epithelialization and matrix formation. 

LRRFIP-1 is important in the regulation of TLR4-mediated tissue inflammation through inhibition of the IRAK cellular cascade and inactivation of the NFKB pathway, with the resulting loss or down regulation of inflammatory cytokine secretion that may affect wound repair, including IL-8, IL-6, TNFα, and IL-2 [15]. LRRFIP-1’s role in TLR signaling and innate inflammatory responses has also been linked to its interactions with a negative regulator of wound healing, Flii, where LRRFIP-1 competes with Flii for the binding to the MyD88 and hence counteracts the effects of Flii on inflammation signaling pathways [11]. Our studies have investigated the role of Flii as a negative regulator of healing in both acute and chronic wound pathologies and most recently in the inflammatory skin condition psoriasis [2,16,17,18]. We have also shown that application of Flii-neutralizing antibodies to wounds in vivo neutralizes extracellular Flii, resulting in improved wound re-epithelialization, decreased tissue inflammation, increased cellular proliferation and migration, and significantly decreased early scar formation in both small and large animal models of wound repair [2,17]. In this study, two doses of rLRRFIP-1 treatment were tested, with both showing improvements in wound healing. Our in vitro experiments showed increased keratinocyte and fibroblast proliferation in response to rLRRFIP-1 treatment and these findings were supported by our in vivo data showing that rLRRFIP-1 increased keratinocyte proliferation in the neoepidermis of day 3 and day 7 wounds and resulted in a significant reduction in Flii levels in the wounds in vivo. Similarly, rLRRFIP-1 decreased inflammation in the wounds. Previous studies have shown that downregulating Flii levels, either genetically or by using Flii-neutralizing antibodies, in chronic diabetic wounds of mice results in significantly decreased TLR4 expression [19]. We now show, similarly, that acute wounds treated with rLRRFIP-1 show significantly decreased TLR4 expression and concurrent decreased inflammatory cell infiltrate, including both neutrophils and macrophages, and show no effect on wound angiogenesis. 

Since collagen deposition is one of the key cellular processes affecting wound remodeling, the effect of rLRRFIP-1 on TGF-β signaling and regulation of collagen levels is of great significance to understanding the mechanism by which LRRFIP-1 affects wound repair. Studies to date have shown that reducing Flii expression, both genetically and using Flii neutralizing antibodies, downregulates collagen I synthesis, with authors hypothesizing that slower, more organized collagen fiber production leads to a better physiological outcome [2,17]. In this study, we show that increased exogenous rLRRFIP-1 levels significantly decrease the pro-scaring TGF-β1 while increasing TGF-β3 levels, suggesting a possible anti-scarring effect of LRRFIP-1 on wound repair. Unlike the effects of Flii-neutralizing antibodies on decreasing collagen deposition, we found that rLRRFIP-1 treatment increased the production of total collagen in wounds in vivo. Collagen synthesis is required for a wound to heal and its contraction is an important prerequisite of a healing wound. Despite the decrease in TGF-β signaling, rLRRFIP-1-treated wounds showed increased total collagen levels, suggesting that LRRFIP-1 effects on collagen deposition during wound healing may be independent of Flii. Studies to date have not identified if exogenous LRRFIP-1 can have an intracellular effect or if there is a cell surface receptor for LRRFIP-1 ligand. However, as with other proteins containing the LRR domain, it is likely that LRRFIP-1 may be involved in other signaling pathways yet to be identified.

## 4. Materials and Methods

### 4.1. Animal Studies

Mice were maintained according to Australian Standards for Animal Care under protocols approved by the Child, Youth, and Women’s Health Service Animal Ethics Committee (AEC889/12/13; 4 October 2011). All studies were performed in female wild-type mice of BALB/c background. 

### 4.2. Murine Surgery Techniques, Histology, and Immunohistochemistry

Two full-thickness, 1-cm incisions were created on the backs of 12-week-old BALB/c mice using protocols previously described [20]. Digital photographs of the wounds were taken for macroscopic analysis of wound healing (wound area and gape) and wounds were harvested at days 3, 7, and 14 post-wounding [20]. Unwounded adult BALB/c mouse skin was also collected for comparison of LRRFIP-1 expression. To a subset of mice, a single intradermal injection of 100 µL of recombinant LRRFIP-1 protein (human recombinant protein P01 #000-11508, Sapphire Biosciences, Redfern, NSW, Australia) (1 or 10 µg/mL) or PBS control was injected into the wound margins of incisional wounds created as described above and left to heal for 7 days before harvesting and processing. Histological sections (4 µm) were stained with haematoxylin-eosin for microscopic analysis of wound healing (wound area, wound gape, and wound re-epithelialization), Masson’s trichrome for total collagen analysis following established protocols [2,17], or subjected to routine immunohistochemistry protocols. 

Immunohistochemistry protocols utilized the following antibodies: LRRFIP-1 rabbit polyclonal (Bioss Antibodies BS-12439R, Woburn, MA, USA) (1 µg/mL), proliferating cell nuclear antigen (PCNA) (#sc-56, Santa Cruz Biotechnology, Dallas, TX, USA) (2 µg/mL), flightless I (Flii) (#sc-21716, Santa Cruz Biotechnology) (1 µg/mL), vimentin (V9) (#sc-6260, Santa Cruz Biotechnology) (1 µg/mL), keratin-10 Ab-2 (#DE-K10, Thermo Fisher Scientific, Scoresby, NSW, Australia) (1 µg/mL), CD31 rabbit polyclonal (#ab28364, Abcam, Cambridge, UK) (1 µg/mL), TLR4/CD284 (#IMG-5794, Imgenex, CA, USA) (1 µg/mL), NIMP-R14 (#sc-59338, Santa Cruz Biotechnology) (2 µg/mL), F4/80 rat monoclonal (#ab6640, Abcam) (1 µg/mL), TGF-β1 (#sc-146-G, Santa Cruz Biotechnology) (1 µg/mL), TGF-β3 (#sc-166861, Santa Cruz Biotechnology) (1 µg/mL), and species-specific secondary antibodies Alexa Flour (#A11008, Invitrogen, Melbourne, VIC, Australia) (2 µg/mL) and Alexa Flour (#A11020, Invitrogen) (2 µg/mL) following established protocols [20,21]. Image analysis was performed using AnalySIS software package version 7.0 (Soft Imaging System GmbH, Munster, Germany). Masson’s trichrome stained sections were used to quantify the total collagen content within the wounds using the colour threshold function on the Image-Pro Plus Software System version 7.0 as previously described [17]. 

### 4.3. Cell Proliferation Assay

Cell proliferation assays were performed on human keratinocytes (HaCats) and human foreskin fibroblasts (HFFs) cell lines using the metabolic substrate WST-1 according to the manufacturer’s protocols (Roche Applied Science, Munich, Germany) and 24 h following treatment with rLRRFIP-1 (50 and 100 ng/mL) or PBS control following established protocols [2]. Additionally, the effect of rLRRFIP-1 treatment (50 and 100 ng/mL) was investigated by fixing sub-confluent cells in 4% PFA and co-staining with PCNA and DAPI following established protocols [20,21].

### 4.4. Coimmunoprecipitation

HaCaTs and HFFs were used in the immunoprecipitation experiment. Briefly, cells were grown to confluence, wounded as described in the scratch assay, and allowed to migrate for 2.5 h before being washed in PBS and lysed on ice with cell lysis buffer (50 mM Tris, 1 Mm EDTA, 50 mM NaCl, 0.1% Triton X-100) supplemented with Protease Inhibitor Complete Mini cocktail tablet (Roche, Sydney, NSW, Australia). The cells were precleared by centrifugation and incubated with 1 µg/1 µL Flii or LRRFIP-1 antibodies and protein G-agarose beads (Invitrogen) overnight at 4 °C. The immunoprecipitates were collected by centrifugation, and the pellets washed with lysis buffer and stored in 2× sample buffer prior to standard Western blotting analysis as previously described [22]. Western blots were probed with either Flii (1 µg/mL) or LRRFIP-1 (2 µg/mL) antibody and whole cell lysate was used as a control. 

### 4.5. Statistical Analysis

Statistical differences were determined using the Student’s *t*-test or an analysis of variance. For data not following a normal distribution, the Mann–Whitney U test was performed. A *p* value of <0.05 was considered significant.

## 5. Conclusions

In summary, LRRFIP-1 is produced by keratinocytes and fibroblasts and its expression is elevated in response to wounding, peaking in the early inflammatory phase of wound healing. While the exact mechanism by which LRRFIP-1 affects inflammatory responses and collagen deposition during wound healing remains unknown and whether those mechanisms are independent of Flii remains to be investigated, these studies clearly demonstrate the therapeutic potential of rLRRFIP-1 for the treatment of acute wounds and a functional significance on cellular responses important during wound healing, including cell proliferation, inflammatory responses, and collagen matrix synthesis.

## Supplementary Materials

Supplementary materials can be found at http://www.mdpi.com/1422-0067/19/7/2014/s1.

## Abbreviations

rLRRFIP-1	Recombinant Leucine-Rich Repeat Flightless-Interacting Protein-1
Flii	Flightless I
PCNA	Proliferating Cell Nuclear Antigen
NIMP-R14	Neutrophil Marker Antibody
F4/80	Macrophage Marker Antibody
