# Supplementary figures and images for: Recombinant Leucine-Rich Repeat Flightless-Interacting Protein-1 Improves Healing of Acute Wounds through Its Effects on Proliferation Inflammation and Collagen Deposition
Source: Int J Mol Sci. 2018 Jul 10;19(7):2014. doi: 10.3390/ijms19072014 (PMC6073877; doi:10.3390/ijms19072014)

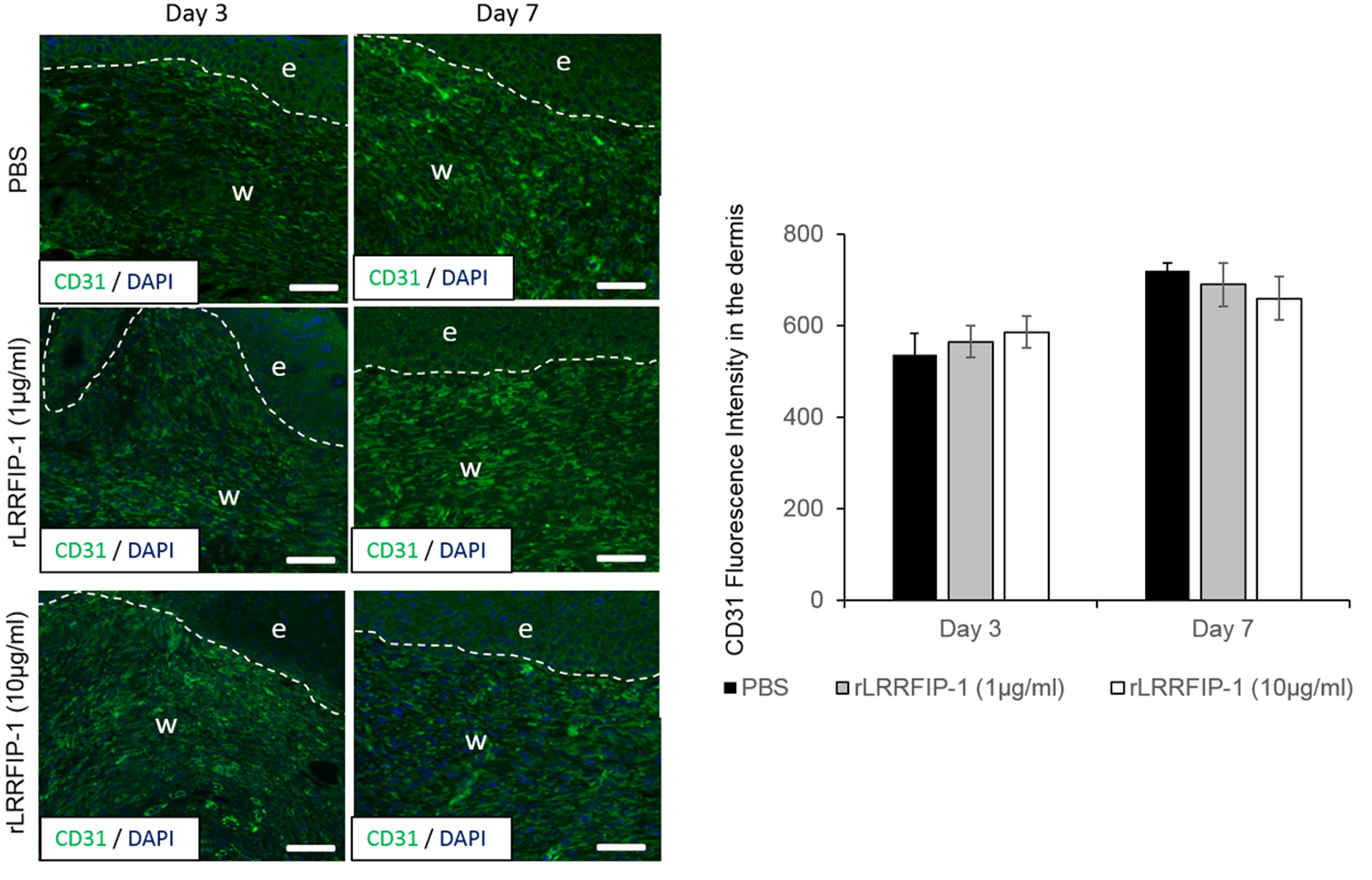

Supplement: Supplementary file 1 [file ijms-19-02014-s001.zip › Suplementary Figure 1.tif]

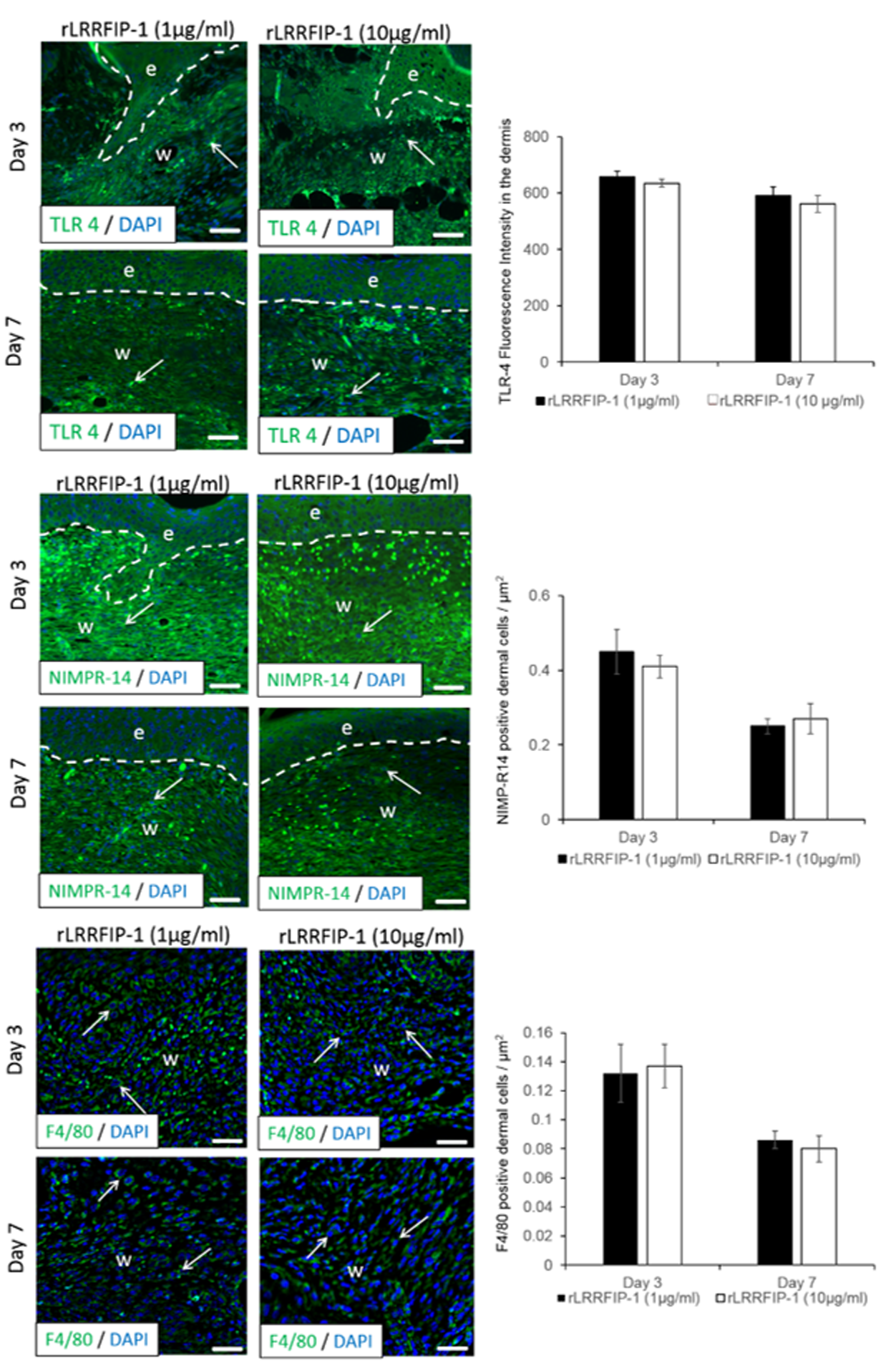

Supplement: Supplementary file 1 [file ijms-19-02014-s001.zip › Suplementary Figure 2.tif]

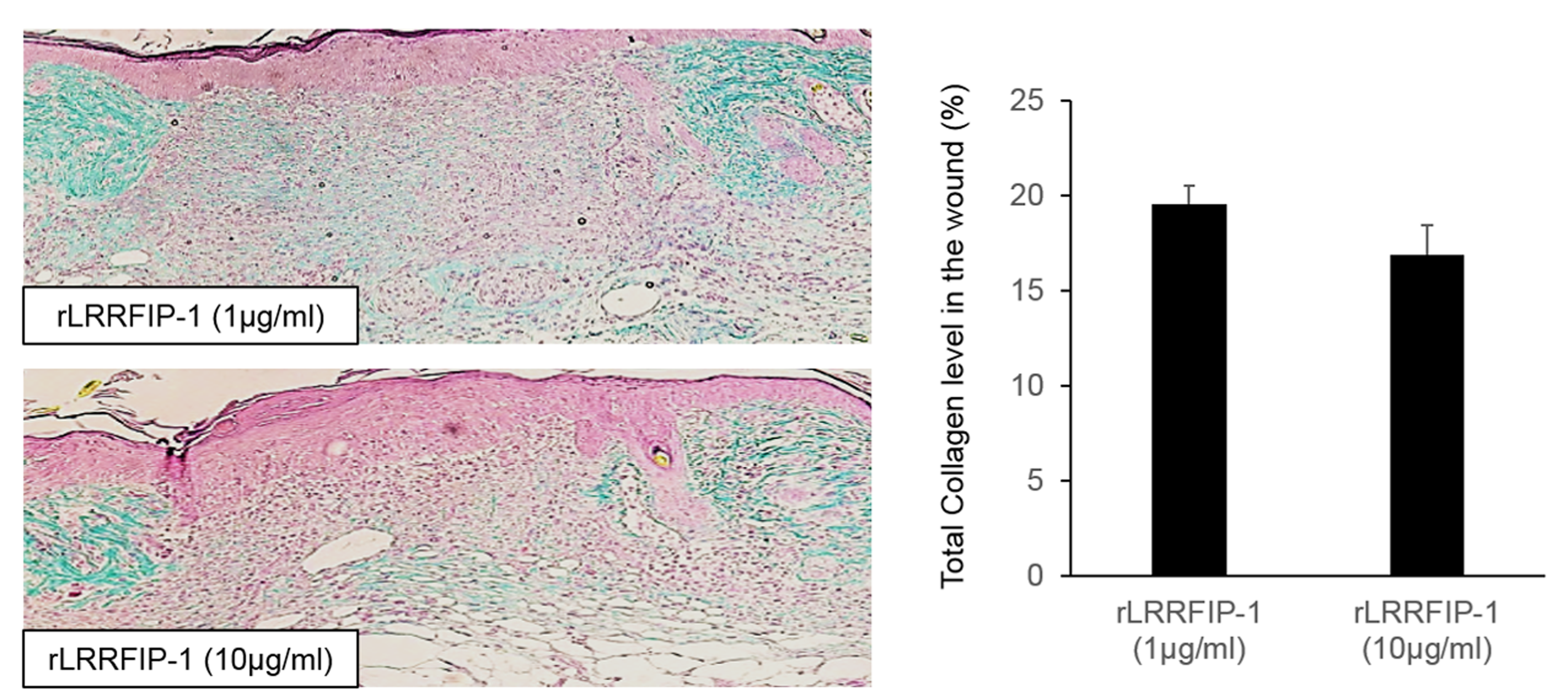

Supplement: Supplementary file 1 [file ijms-19-02014-s001.zip › Suplementary Figure 3.tif]
